# Atherosclerosis Prevention in Adolescents with Obesity: The Role of Moderate–Vigorous Physical Activity

**DOI:** 10.3390/ijerph192315537

**Published:** 2022-11-23

**Authors:** Antonio Videira-Silva, Luis B. Sardinha, Helena Fonseca

**Affiliations:** 1 Pediatric University Clinic, Faculty of Medicine, Universidade de Lisboa, 1649-035 Lisbon, Portugal; 2 CIDEFES (Centro de Investigação em Desporto, Educação Física, Exercício e Saúde), Universidade Lusófona, 1749-024 Lisbon, Portugal; 3 Exercise and Health Laboratory, CIPER, Faculty of Human Kinetics, Universidade de Lisboa, 1649-004 Lisbon, Portugal; 4 Pediatric Obesity Clinic, Department of Pediatrics, Hospital de Santa Maria, 1649-035 Lisbon, Portugal

**Keywords:** adolescents, overweight, obesity, carotid intima–media thickness, cardiorespiratory fitness, physical activity

## Abstract

Carotid intima–media thickness (cIMT) is a subclinical marker of atherosclerotic development, which is impaired in adolescents with obesity. This study aimed to analyze the impact of physical activity (PA), cardiorespiratory fitness (CRF), body mass index (BMI), and body composition changes on the cIMT of adolescents with obesity. Longitudinal data (6 months) from adolescents aged 12–18 years, with a BMI ≥97th percentile, previously recruited for the non-randomized controlled trial PAC-MAnO (Clinicaltrials.gov-NCT02941770) were analyzed using partial correlations controlling for sex and pubertal status and multiple regressions. A total of 105 adolescents (51.4% girls, 86.7% Caucasian), 14.8 ± 1.8 years old, with a BMI z-score of 3.09 ± 0.74 were included. Total body fat mass (TBFM) (F(1,91) = 23.11, *p* < 0.001), moderate–vigorous PA (MVPA) (F(1,91) = 7.93, *p* = 0.0006), and CRF (mL/kg/min) (F(1,90) = 19.18, *p* < 0.001) predicted cIMT variance with an R^2^ of 0.24, 0.09, and 0.23, respectively. MVPA changes showed a high correlation with CRF variation (r(91) = 0.0661, *p* < 0.001). This study suggests that although cIMT is impaired in overweight adolescents, improvements in TBFM, MVPA, and CRF are associated with cIMT improvement. Although both energy intake and MVPA may influence TBFM, MVPA plays the most relevant role in cIMT development due to its direct association with CRF.

## 1. Introduction

Obesity in adolescence has been associated with several adverse health consequences [1], which are increasing in prevalence along with the increased prevalence [2] and severity of adolescent obesity [3].

Adolescent obesity is associated not only with increased disability [4], but also with increased cardiovascular morbidity and mortality in adulthood [5].

Carotid artery intima–media thickness (cIMT) is a primary marker for atherosclerotic cardiovascular disease (CVD) [6] and reflects the endothelial structure preceding the formation of atheromatous plaque [7,8]. Although cIMT may be influenced by genetic factors [9], obesity (in particular central obesity), as well as the presence of other CVD risk factors, may play a central role in cIMT development [10,11,12,13]. On the other hand, cardiorespiratory fitness (CRF) and physical activity (PA) have been acknowledged as possible antagonistic modifiable factors that may play a crucial role in cIMT reversion in adolescents with obesity [14]. It has been suggested that the beneficial effect that PA may have on the endothelial structure and function is related to the increase in blood flow and shear-stress-dependent mechanisms that influence vasodilators, such as nitric oxide (NO) availability [15,16,17], independently of other CVD risk factors [18].

It is crucial not only to identify subclinical atherosclerotic development early on but also investigate the role that modifiable risk factors, such as CRF and PA, may play in this process.

To date, few prospective studies have investigated the impact of body mass index (BMI), body composition, PA, and CRF changes on cIMT in adolescents with obesity [19,20]. This study adds to the existing literature by using objective assessments of the main variables under study, contributing to the consistency of knowledge regarding atherosclerosis prevention in adolescents with obesity.

This study aimed to explore possible associations between 6-month changes in PA, CRF, BMI, and body composition with cIMT changes in adolescents with obesity.

We hypothesized that changes in BMI and body fat mass would be positively associated with cIMT and, conversely, that increased CRF, as well as moderate and vigorous PA levels, would be inversely associated.

## 2. Materials and Methods

### 2.1. Participants

Data from adolescents aged 12–18 years, with a BMI over the 97th percentile (for gender and age) [21], previously recruited for the non-randomized controlled trial PAC-MAnO (the effect of a PA consultation in the management of adolescent overweight) were used in this study. The PAC-MAnO project is registered with Clinicaltrials.gov (NCT02941770) and its study protocol can be found elsewhere [22].

Participants with major pathologies (other than obesity or related comorbidities), mental disorders, smoking habits, conditions leading to an inability to perform regular PA, or who were involved in other weight loss programs were excluded.

Informed assent/consent was obtained from all participants and their respective caregivers.

This study was approved by the Ethics Committee of the Faculty of Medicine of the University of Lisbon, Portugal (271/2016) and is in accordance with the 1964 Helsinki declaration and its later amendments or comparable ethical standards.

### 2.2. Measurements

#### 2.2.1. Anthropometric and Body Composition Assessment

Height, registered to the nearest 0.1 cm, was assessed in the anthropometric position, using the Frankfurt plan, without shoes, and after an expiratory phase (height stadiometer, SECA 217, Hamburg, Germany).

Bodyweight, measured to the nearest 0.1 kg, was assessed with the subject wearing as little clothing as possible and without shoes or socks (bioelectrical impedance scale InBody 230, Seoul, Korea).

BMI was calculated as body weight in kilograms divided by the square of height in meters (BMI = weight (kg)/height2 (m)). The BMI z-score was further calculated according to the World Health Organization reference [23] using an AnthroPlus calculator (version 1.0.4, WHO).

Waist circumference (WC) was measured with the subject standing, 1 cm above the iliac crest, at the end of a regular expiration (circumference measuring tape, SECA 203, Hamburg, Germany). The Waist-to-Height ratio (WHtR) was further calculated (WHtR = WC/Height).

Body composition was assessed by Dual-energy X-ray absorptiometry (DXA) (Explorer W, Hologic; Waltham, MA, USA) and analyzed using the equipment’s software (QDR 12.4, Waltham, MA, USA). A DXA exam was performed following the National Health and Nutrition Examination Survey (NHANES) protocol [24]. Total body fat mass (TBFM), trunk fat mass (Trunk FM), and fat-free and bone-free mass (FBFM) were considered measures of interest. Relative body fat mass (BFM) and muscle mass (MM) were calculated as TBFM and FBFM divided by body weight, respectively, and are expressed as a percentage (%).

#### 2.2.2. Clinical Assessments

Pubertal status was objectively assessed by a pediatrician and categorized according to Tanner’s stages.

Resting blood pressure was measured in the right arm with an appropriately sized cuff after five minutes of rest in the sitting position. The measurement was performed three times and the average of the systolic blood pressure (SBP) and the diastolic blood pressure (DBP) was recorded (digital sphygmomanometer, CAS 9302S, CAS Medical Systems, Branford, CT, USA).

Cardiorespiratory fitness (CRF), i.e., oxygen uptake during peak exercise (VO_2_ peak), was directly determined with a Gas analyzer (K4 b2, Cosmed, Rome, Italy) during a submaximal exercise test in a cycle ergometer (electronically braked cycle ergometer, Monark 839 Ergomedic, Monark, Vansbro, Sweden). The initial workload and increments were 40W for girls and 50W for boys. Heart rate was registered continuously with a cardiofrequencimeter (Polar Vantage NV, Polar Electro Oy, Kempele, Finland). A heart rate ≥ 85% of the theoretical maximal heart rate [25], failure to maintain a frequency of at least 30 revolutions/min, and a subjective judgment by the observer that the adolescent was exhausted were considered to be criteria to stop the test [26]. Measured VO_2_ peak (mL/min) was further adjusted for body weight (mL/kg/min) and additionally used in the analyses.

Carotid intima–media thickness (cIMT) was measured with an ultrasound imager using a 13 MHz probe (MyLab One, Esaote, Genoa, Italy). cIMT was defined as the distance between the lumen–intima and the media–adventitia interfaces. The measurement was performed in the longitudinal plane on the right carotid artery and in accordance with previously validated radiofrequency-based tracking of the arterial wall that allows for a real-time determination of the common carotid far-wall thickness (QIMT^®^) with high spatial and temporal resolution. cIMT was automatically measured, and distension curves were acquired within a CCA 1.59-cm region of interest, approximately 1 cm proximal to the carotid bifurcation. Mean cIMT was used in the analyses and the cIMT/diameter ratio was further calculated.

Girls at the time of the clinical and anthropometric/body composition assessments were not menstruating.

#### 2.2.3. Physical Activity Assessment

PA was assessed with accelerometers (ACTIGRAPH GT3X, Pensacola, Florida, USA), programmed to use a 5-s cycle, during at least one weekend day and two weekdays. Only days with more than 480 min (8 h) registered were considered in the analysis. Activities between 0 and 149 counts/minute were considered to be sedentary activities, activities between 150 and 499 counts/minute were considered to be light physical activities (LPA), activities between 500 and 3999 counts/minute were considered to be moderate physical activities (MPA), and activities with more than 4000 counts/minute were considered to be vigorous physical activities (VPA) [27]. The daily average of sedentary time, LPA, MPA, and VPA was calculated and used in the analysis.

### 2.3. Statistical Analysis

Data were analyzed using the IBM SPSS statistics package (IBM SPSS statistics, version 26.0, IBM, New York, NY, USA). An independent sample *t*-test/Mann–Whitney U test and a Qui-squared test were used to analyze baseline differences between girls and boys for continuous and categorical variables, respectively. Overtime within-group and between-group differences were analyzed with a paired sample *t*-test/Wilcoxon test and Generalized Estimating Equations. Associations among variables of interest were analyzed with nonparametric partial correlations controlling for sex and pubertal status (i.e., Tanner stage) using SPSS syntax commands and multiple linear regressions (stepwise and enter methods). A *p*-value of ≤0.05 was considered statistically significant.

## 3. Results

Results are reported according to the Consolidated Standards of Reporting Trials (CONSORT) recommendations for randomized clinical trials [28].

Data from 105 adolescents (51.4% girls, 86.7% Caucasian) aged 14.8 ± 1.8 years, with a BMI of 34.60 ± 5.25 and a BMI z-score of 3.09 ± 0.74, who completed 6-month assessments of the PAC-MAnO project collected between September 2016 and June 2019 were analyzed. No overtime changes in pubertal status were observed during the 6 months (data not reported).

### 3.1. Sex Differences

At baseline, girls showed a higher pubertal status (75.9% in Tanner’s stage 5 vs. 33.3%, *p* < 0.001) and cIMT/diameter ratio (7.71 [95% CI −3.2 to 21.7], *p* = 0.0008) and a lower weight (−8.8 kg [95% CI −16.0 to −1.6], *p* = 0.0018), height (−6.4 cm [95% CI −9.2 to −3.5], *p* < 0.001), BMI z-score (−0.38 [95% CI −0.66 to −0.09], *p* = 0.0010), WC (−6.9 cm [95% CI −11.8 to −2.0], *p* = 0.0007), MM (−1.5 % [95% CI −2.8 to −0.3], *p* = 0.0019), FBFM (−6.6 kg [95% CI −1.0 to −3.2], *p* < 0.001), and VO_2_ peak (−338 mL/min [95% CI −487 to −189], *p* < 0.001; −1.46 mL/kg/min [95% CI −2.61 to −0.31], *p* = 0.0013) compared with boys. Baseline characteristics are presented in Table 1.

No overtime differences were found between girls and boys. Overtime within-group and between-group changes are presented in Table 2.

### 3.2. Correlation Analyses

In order to enable robust correlation analyses, girls and boys were analyzed together. However, because statistically significant differences between sexes were identified at baseline, nonparametric correlation analyses controlling for sex and pubertal status (i.e., Tanner stage) were performed.

cIMT variation was positively correlated with overtime changes in BMI z-score (r(91) = 0.0296, *p* = 0.0023), WHtR (*r(91)* = 0.0323, *p* = 0.0013), TBFM (*r(90)* = 0.0306, *p* = 0.0018), and Trunk FM (r(90) = 0.0301, *p* = 0.0021) and negatively correlated with MPA (*r(91)* = −0.470, *p* < 0.001), VPA (r(91) = −0.331, *p* = 0.0017), MVPA (r(91) = −0.515, *p* < 0.001), and VO_2_ peak (both absolute and relative) (*r(89)* = −0.410, *p* = 0.0001; *r(89)* = −0.435, *p* = 0.0001).

cIMT/diameter ratio variation was positively correlated with changes in WHtR (*r(91)* = 0.0306, *p* = 0.0019) and Trunk FM (*r(90)* = 0.0287, *p* = 0.0028) and negatively correlated with MM (*r(90)* = −0.265, *p* = 0.0042), MPA (*r(91)* = −0.300, *p* = 0.0021), VPA (*r(91)* = −0.446, *p* < 0.001), MVPA (*r(91)* = −0.412, *p* = 0.0001), and VO_2_ peak (both absolute and relative) (*r(89)* = −0.303, *p* = 0.0020; *r(89)* = −0.352, *p* = 0.0006).

Overtime changes in MVPA showed a robust negative correlation with TBFM (*r(91)* = −0.568, *p* < 0.001) and a positive correlation with VO_2_ peak (both absolute and relative) (*r(89)* = 0.0525, *p* < 0.001; *r(89)* = 0.0661, *p* < 0.001). Energy intake was positively correlated with TBFM (*r(85)* = 0.0427, *p* < 0.001), but not with cIMT or cIMT/diameter ratio (*r(85)* = 0.0224, *p* = 0.0066; *r(85)* = 0.0224, *p* = 0.0066) (data not shown). Correlation analyses are presented in Table 3.

### 3.3. Regression Analyses

According to multiple regression analysis, TBFM variation was the best anthropometric/body composition predictor of both cIMT and cIMT/diameter ratio changes (F(1,91) = 23.11, *p* < 0.001; F(1,91) = 16.93, *p* < 0.001) with an *R*^2^ of 0.24 and 0.18, respectively. VO_2_ peak (mL/kg/min) was the best clinical predictor of both cIMT and cIMT/diameter ratio evolution (F(1,90) = 19.18, *p* < 0.001; F(1,90) = 13.73, *p* < 0.001) with an *R*^2^ of 0.23 and 0.17, respectively. Regarding PA behavior, changes in MVPA and VPA predicted cIMT (F(1,92) = 7.93, *p* = 0.0006) and cIMT/diameter ratio evolution F(1,92) = 12.65, *p* = 0.0001), with an *R*^2^ of 0.09 and 0.14, respectively (Table 4).

## 4. Discussion

This study aimed to explore possible associations between 6-month changes in PA, CRF, BMI, and body composition with cIMT changes in adolescents with obesity.

It was hypothesized that changes in BMI and BFM would be positively associated with cIMT since, according to the literature, cIMT is associated with high adiposity, dyslipidemia, raised blood pressure, insulin resistance, and pro-inflammatory markers, which are commonly present in adolescents with obesity [11,29]. This study shows a positive correlation among BMI/BMI z-score, BFM/TBFM, Trunk FM, and cIMT changes over time (Table 3), which is in line with the literature [29,30] showing also that changes in TBFM were the best anthropometric/body composition predictor of cIMT and cIMT/diameter ratio, predicting 24 and 18% of their variance (Table 4).

Contrary to what has been suggested by cross-sectional associations [14,31,32], changes in central adiposity (here assessed by Trunk FM and WHtR) were not the best predictors of cIMT variations in the present study. This unexpected result may be associated with the fact that both Trunk FM and WHtR are undifferentiated measures of central adiposity, not enabling the distinction between visceral and subcutaneous adipose tissue. A high proportion of visceral compared with subcutaneous adipose tissue is associated with higher insulin resistance and triglyceride levels and decreased HDL-C and adiponectin levels [33], increasing the risk of metabolic syndrome development [34] and, consequently, of cIMT [13,35,36]. It is worth noting that, in the pediatric age range, due to growth and maturity, WHtR may be more reliable than WC alone for tracking changes in central adiposity [37]; therefore, WHtR was used in the analysis.

It was further hypothesized that overtime changes in CRF, as well as in MPA and VPA, would be inversely associated with cIMT variation. Indeed, CRF (VO_2_ peak, mL/kg/min) not only showed an inverse correlation with cIMT and cIMT/diameter ratio but was the second-best predictor of cIMT and cIMT/diameter ratio, explaining 23 and 17% of their variance, respectively. These results are in line with the results reported by Farpour-Lambert et al. [19] and Park et al. [20] suggesting that major improvements in CRF are linked to greater improvements in cIMT. It has to be highlighted that only relative CRF (mL/kg/min) was shown to predict cIMT and cIMT/diameter ratio variation, which can be explained by the inclusion of “weight” (which was shown to be positively correlated with cIMT) in the equation.

As initially hypothesized, changes in both MPA and VPA (and MVPA) were negatively associated with changes in cIMT. However, MVPA showed a lower capacity to predict cIMT and cIMT/diameter ratio variance compared with CRF, suggesting that CRF may moderate the relationship between MVPA and cIMT [14]. In fact, although Meyer et al. [38] reported a decrease in cIMT after a 6-month exercise intervention, there is no consensus that PA levels or intensities are negatively associated with cIMT independently of changes in CRF [14].

It should be additionally noted that changes in VPA showed a stronger correlation with cIMT/diameter ratio variation (*r(91)* = −0.446, *p* < 0.001) than with cIMT (*r(91)* = −0.311, *p* = 0.0017). This result may be explained, in part, by an increase in artery diameter as a consequence of arteriogenesis-related mechanisms induced by VPA [39]. Although cIMT per se may be a reliable indicator of primary atherosclerotic development, artery diameter is positively associated with cIMT [32], which may reflect an adaptive response to the developmental process [14] and not an endothelial structural or functional health impairment. Nevertheless, according to the statistical analyses, no other relevant differences were found regarding the relationship between cIMT and cIMT/diameter ratio and the other variables under study.

The main limitation of this study is the lack of longitudinal information regarding biochemical markers (e.g., glucose and insulin levels, HOMA, TC, LDL-C, HDL-C, TG, ALT, and CRP), which are well-known indicators of cardiovascular health [40], being associated with both BFM and cIMT [6,41,42]. Another possible limitation is the lack of data among girls on the timing of menarche. Nevertheless, there is no consensus that pubertal timing has a significant effect on the vascular structure and function in adults when controlling for pre-pubertal BMI [43]. Despite this limitation, this study contributes to our understanding of the impact of BMI, body composition, PA, and CRF on endothelial structure health among overweight adolescents.

## 5. Conclusions

Although changes in energy intake are positively associated with TBFM variance (the best predictor of cIMT), they are not associated with cIMT. On the other hand, MVPA is not only inversely associated with TBFM (possibly due to its relevant role in energy expenditure) but also positively associated with CRF (the second-best predictor of cIMT). This study clearly shows that although CRF may moderate the relationship between MVPA and cIMT, which can be linked to genetic factors [44], MVPA, as a modifiable factor, should be promoted among overweight adolescents in order to attenuate subclinical atherosclerotic development.

## Figures and Tables

**Table 1:** Participants’ characteristics at baseline.

		Girls (*n* = 54)	Boys (*n* = 51)		Total (*n* = 105)
		*n* (%)		*p*	*n* (%)
Race (Caucasian, *n*)		48 (88.9%)	43 (84.3%)	0.736 ^a^	91 (86.7%)
Tanner stage (*n*)	2	0 (0%)	9 (17.7%)	<0.001 ^a^	9 (8.6%)
	3	5 (9.3%)	13 (25.5%)		18 (17.1%)
	4	8 (14.8%)	12 (23.5%)		20 (19.1%)
	5	41 (75.9%)	17 (33.3%)		58 (55.2%)
		**Mean ± SD**		***p***	**Mean ± SD**
Age (years)		15.1 ± 1.5	14.6 ± 2.0	0.206 ^b^	14.8 ± 1.8
Weight (kg)		89.7 ± 13.2	98.5 ± 22.5	0.018 ^b^	94.0 ± 18.8
Height (cm)		161.3 ± 6.1	167.6 ± 8.4	<0.001 ^b^	164.3 ± 7.9
BMI (kg/m^2^)		34.44 ± 4.33	34.78 ± 6.12	0.740 ^b^	34.23 ± 7.13
BMI z-score		2.91 ± 0.60	3.28 ± 0.83	0.010 ^b^	3.09 ± 0.74
HipC		116.3 ± 8.2	117.6 ± 12.0	0.523 ^b^	117.0 ± 10.2
WC		104.6 ± 10.0	111.5 ± 14.5	0.007 ^b^	108.0 ± 12.9
WHtR		0.65 ± 0.06	0.67 ± 0.08	0.242 ^b^	0.65 ± 0.09
BFM (%)		45.6 ± 5.3	43.4 ± 6.1	0.052 ^b^	45.8 ± 8.7
TBFM (kg)		37.7 ± 9.0	39.2 ± 11.4	0.478 ^b^	38.5 ± 10.2
Trunk FM (kg)		17.7 ± 5.0	18.1 ± 6.0	0.715 ^b^	17.9 ± 5.5
MM (%)		30.1 ± 3.0	31.6 ± 3.5	0.019 ^b^	30.8 ± 3.4
FBFM (kg)		47.1 ± 5.5	53.7 ± 11.0	<0.001 ^b^	49.7 ± 13.0
Sedentary (min/day)		606.9 ± 126.3	627.7 ± 114.6	0.394 ^b^	616.8 ± 120.8
LPA (min/day)		56.5 (51.3)	45.6 (53.0)	0.108 ^c^	55.5 (56.1)
MPA (min/day)		27.8 (31.8)	31.2 (25.8)	0.883 ^c^	30.3 (29.7)
VPA (min/day)		3.6 (5.0)	5.1 (6.7)	0.149 ^c^	4.2 (6.6)
MVPA (min/day)		35.5 (33.4)	34.4 (23.1)	0.793 ^c^	34.9 (31.0)
VO_2_ (mL/min)		1788 ± 254	2126 ± 457	<0.001 ^b^	1950 ± 401
VO_2_ (mL/kg/min)		20.07 ± 2.87	21.54 ± 2.92	0.013 ^b^	20.78 ± 2.98
SBP (mmHg)		119.6 ± 11.0	121.9 ± 11.3	0.306 ^b^	120.0 ± 10.0
DBP (mmHg)		65.5 ± 9.0	65.9 ± 9.8	0.816 ^b^	66.0 ± 11.0
cIMT (μm)		668 ± 136	661 ± 105	0.778 ^b^	651 ± 174
cIMT/diameter ratio		98.66 (39.12)	90.95 (24.01)	0.008 ^c^	96.4 (29.4)

BFM, body fat mass; BMI, body mass index; cIMT, carotid intima–media thickness; DBP, diastolic blood pressure; FBFM, fat-free and bone-free mass; HipC, hip circumference; LPA, light physical activity; MM, muscle mass; MPA, moderate physical activity; MVPA, moderate–vigorous physical activity; SBP, systolic blood pressure; TBFM, total body fat mass; Trunk FM, trunk fat mass; VPA, vigorous physical activity; WC, waist circumference; WHtR, waist–height ratio. ^a,b,c^ Between-group differences were analyzed with a Qui-squared, independent sample *t*-test and a Mann–Whitney U test, respectively. For the Mann–Whitney U test, median (interquartile range) values are presented.

**Table 2:** Overtime changes from baseline to 6 months by sex.

	Girls (*n* = 54)			Boys (*n* = 51)			Girls * Boys	
	Δ	95% CI	*p*	Δ	95% CI	*p*	*β* (95% CI)	*p*
Weight (kg)	0.2 ± 4.8	−1.2, 1.5	0.799 ^a^	1.6 ± 6.6	−0.4, 3.6	0.115 ^a^	1.9 (−1.6, 5.3)	0.292
Height (cm)	0.5 ± 0.8	0.3, 0.8	<0.001 ^a^	1.6 ± 1.4	1.1, 2.0	<0.001 ^a^	0.8 (−0.2, 1.9)	0.112
BMI (kg/m^2^)	−0.15 ± 1.78	−0.65, 0.34	0.537 ^a^	−0.02 ± 2.0	−0.63, 0.58	0.938 ^a^	0.38 (−0.61, 1.37)	0.452
BMI z-score	−0.08 ± 0.27	−0.16, −0.01	0.035 ^a^	−0.09 ± 0.31	−0.19, 0.00	0.055 ^a^	0.02 (−0.13, 0.17)	0.773
HipC (cm)	0.2 ± 4.7	−1.2, 1.5	0.813 ^a^	−0.8 ± 4.1	−2.1, 0.5	0.243 ^a^	−1.5 (−4.1, 1.0)	0.239
WHtR	−0.01 ± 0.03	−0.01, 0.00	0.255 ^a^	−0.01 ± 0.03	−0.02, −0.00	0.023 ^a^	−0.00 (−0.00, 0.01)	0.842
BFM (%)	−0.6 ± 2.7	−1.4, 0.2	0.116 ^a^	−1.3 ± 3.5	−2.4, −0.2	0.020 ^a^	−1.0 (−2.6, 0.6)	0.204
TBFM (kg)	−0.5 ± 3.4	−1.6, 0.5	0.309 ^a^	−0.4 ± 5.2	−2.2, 1.3	0.609 ^a^	−0.8 (−4.0, 2.5)	0.651
Trunk FM (kg)	−0.8 ± 1.9	−1.4, −0.2	0.010 ^a^	−1.6 ± 2.6	−2.5, −0.7	0.001 ^a^	−1.4 (−3.0, 0.3)	0.117
MM (%)	0.3 ± 1.8	−0.2, 0.8	0.226 ^a^	0.8 ± 2.0	0.2, 1.4	0.011 ^a^	0.7 (−0.3, 1.6)	0.158
FBFM (kg)	0.4 ± 1.6	−0.1, 0.9	0.139 ^a^	2.0 ± 2.4	1.2, 2.9	<0.001 ^a^	1.0 (−1.0, 2.9)	0.327
Sedentary (min/day)	5.2 ± 127.0	17.6, −30.1	0.767 ^a^	−44.4 ± 97.5	−74.8, −14.0	0.005 ^a^	−42.7 (−88.1, 2.7)	0.065
LPA (min/day)	13.0 (29.2)	1.8, 23.6	0.001 ^b^	8.3 (35.4)	−10.1, 22.0	0.133 ^b^	−0.1 (−0.3, 0.2)	0.630
MPA (min/day)	10.8 (21.2)	12.1, 29.7	<0.001 ^b^	19.8 (34.5)	17.7, 32.8	<0.001 ^b^	−0.1 (−0.4, 0.2)	0.387
VPA (min/day)	4.9 (8.7)	3.9, 9.1	<0.001 ^b^	6.4 (10.8)	7.0, 12.4	<0.001 ^b^	−0.1 (−0.5, 0.4)	0.781
MVPA (min/day)	14.4 (27.0)	16.8, 38.0	<0.001 ^b^	31.0 (42.8)	26.4, 43.6	<0.001 ^b^	−0.1 (−0.4, 0.2)	0.478
VO_2_ (mL/min)	58.9 ± 93.9	31.4, 86.5	<0.001 ^a^	95.5 ± 138.6	47.9, 143.1	<0.001 ^a^	−9.3 (−103.3, 84.7)	0.846
VO_2_ (mL/kg/min)	0.84 ± 1.79	0.32, 1.37	0.002 ^a^	0.85 ± 2.27	0.07, 1.63	0.034 ^a^	−0.2 (−1.3, 1.0)	0.751
SBP (mmHg)	−5.0 ± 12.0	−8.8, −1.3	0.010 ^a^	−1.5 ± 13.5	−6.2, 3.1	0.503 ^a^	2.9 (−2.5, 8.3)	0.294
DBP (mmHg)	−1.0 ± 8.0	−3.5, 1.5	0.432 ^a^	−2.3 ± 9.0	−5.4, 0.8	0.142 ^a^	−2.0 (−5.8, 1.8)	0.295
cIMT (μm)	−27 ± 80	−50.6, −3.9	0.023 ^a^	−34 ± 104	−70, 1	0.054 ^a^	−16.3 (−56.7, 24.3)	0.432
cIMT/diameter ratio	−6.45 ± 24.6	−17.99, −4.49	0.002 ^b^	−7.59 (19.3)	−17.94, −1.65	0.020 ^b^	−0.01 (−0.11, 0.09)	0.837

BFM, body fat mass; BMI, body mass index; cIMT, carotid intima–media thickness; DBP, diastolic blood pressure; FBFM, fat-free and bone-free mass; HipC, hip circumference; LPA, light physical activity; MM, muscle mass; MPA, moderate physical activity; MVPA, moderate–vigorous physical activity; SBP, systolic blood pressure; TBFM, total body fat mass; Trunk FM, trunk fat mass; VPA, vigorous physical activity; WHtR, waist–height ratio. ^a,b^ Overtime within-group differences were analyzed with a paired sample *t*-test and Wilcoxon’s test, respectively. βs indicate significant sex-by-time interactions. Unstandardized βs adjusted for Tanner stage.

**Table 3:** Partial correlations controlled for sex and Tanner stage.

Δ	Weight	BMI	BMIz	HipC	WHtR	BFM	TBFM	Trunk FM	MM	FBFM	Sed	LPA	MPA	VPA	MVPA	VO_2_	VO_2_	SBP	DBP	cIMT	cIMT/diam
																(mL/min)	(mL/kg/min)				
Weight	1																				
BMI	0.956 §	1																			
BMIz	0.908 §	0.942 §	1																		
HipC	0.831 §	0.784 §	0.797 §	1																	
WHtR	0.711 §	0.718 §	0.778 §	0.657 §	1																
BFM	0.747 §	0.756 §	0.752 §	0.649 §	0.654 §	1															
TBFM	0.896 §	0.873 §	0.800 §	0.752 §	0.701 §	0.767 §	1														
Trunk FM	0.763 §	0.784 §	0.740 §	0.633 §	0.621 §	0.671 §	0.877 §	1													
MM	−0.734 §	−0.738 §	−0.742 §	−0.637 §	−0.632 §	−0.994 §	−0.756 §	−0.656 §	1												
FBFM	0.246	0.190	0.176	0.133	0.147	0.016	0.007	−0.074	−0.011	1											
Sed	0.203	0.114	0.213	0.272 *	0.177	0.304 *	0.246	0.282 *	−0.278 *	−0.087	1										
LPA	0.034	−0.005	−0.006	−0.255	−0.086	−0.044	0.034	0.001	0.034	−0.034	−0.009	1									
MPA	−0.424 §	−0.465 §	−0.469 §	−0.373 †	−0.432 §	−0.404 †	−0.535 §	−0.528 §	0.403 †	0.245	−0.335 †	−0.041	1								
VPA	−0.363 †	−0.376 †	−0.370 †	−0.262 *	−0.353 †	−0.395 †	−0.432 §	−0.378 †	0.411 §	0.309 *	−0.174	−0.209	0.508 §	1							
MVPA	−0.456 §	−0.496 §	−0.495 §	−0.370 †	−0.451 §	−0.464 §	−0.568 §	−0.539 §	0.462 §	0.280 *	−0.320 *	−0.097	0.939 §	0.720 §	1						
VO_2_ (mL/min)	−0.183	−0.182	−0.218	−0.178	−0.067	−0.313 *	−0.270 *	−0.274 *	0.331 †	0.264 *	−0.195	−0.281 *	0.454 §	0.413 §	0.525 §	1					
VO_2_ (mL/kg/min)	−0.800 §	−0.778 §	−0.766 §	−0.693 §	−0.619 §	−0.745 §	−0.784 §	−0.645 §	0.745 §	−0.071	−0.303 *	−0.174	0.591 §	0.540 §	0.661 §	0.638 §	1				
SBP	0.071	0.092	0.168	0.092	0.166	0.079	0.085	−0.025	−0.098	0.126	−0.239	−0.034	−0.154	−0.002	−0.164	0.026	−0.064	1			
DBP	0.087	0.087	0.021	−0.039	−0.029	0.084	0.102	0.101	−0.064	0.145	0.024	0.008	−0.122	−0.138	−0.201	−0.150	−0.180	0.363 †	1		
cIMT	0.257 *	0.284 *	0.296 *	0.168	0.323 *	0.230	0.306 *	0.301 *	−0.235	−0.041	−0.070	0.011	−0.470 §	−0.311 *	−0.512 §	−0.410 §	−0.435 §	0.267 *	0.336 †	1	
cIMT/diam	0.220	0.211	0.242	0.195	0.306 *	0.251	0.243	0.287 *	−0.265 *	−0.164	−0.025	0.045	−0.300 *	−0.446 §	−0.412 §	−0.303 *	−0.352 †	0.087	0.202	0.676 §	1

BFM, body fat mass; BMI, body mass index; cIMT, carotid intima–media thickness; DBP, diastolic blood pressure; FBFM, fat-free and bone-free mass; HipC, hip circumference; LPA, light physical activity; MM, muscle mass; MPA, moderate physical activity; MVPA, moderate–vigorous physical activity; SBP, systolic blood pressure; TBFM, total body fat mass; Trunk FM, trunk fat mass; VPA, vigorous physical activity; WHtR, waist–height ratio. * *p* < 0.05; † *p* < 0.01; § *p* < 0.05.

**Table 4:** Multiple regression models with carotid intima–media thickness (cIMT) and cITM/diameter ratio as dependent variables.

	cIMT					cIMT/Diameter Ratio				
*Anthropometric/Body composition* ^a^	β	*t*	F	*R* ^2^	*p*	β	*t*	F	*R* ^2^	*p*
Model 1|ΔTBFM	0.49	4.81	23.11	0.24	<0.001	0.43	4.12	16.93	0.18	<0.001
*Clinical* ^a^										
Model 2|ΔVO_2_ max	−0.48	−4.38	19.18	0.23	<0.001	−0.42	−3.71	13.73	0.17	<0.001
Model 3|ΔVO_2_ max, DBP			13.27	0.29	<0.001					
*Physical activity behavior* ^a^										
Model 4|ΔMVPA	−0.30	−0.44	7.93	0.09	0.006					
Model 5|ΔVPA						−0.37	−3.56	12.65	0.14	0.001
*Best fit Model* ^b^										
Model 6|ΔTBFM, ΔVO_2_ max, ΔMVPA			8.55	0.26	<0.001					
Model 7|ΔTBFM, ΔVO_2_ max, ΔVPA								7.36	0.24	<0.001
Model 8|ΔTBFM, ΔVO_2_ max			12.83	0.26	<0.001			9.20	0.20	<0.001
Model 9|ΔTBFM, ΔVPA								10.98	0.23	<0.001

cIMT, carotid intima–media thickness; DBP, diastolic blood pressure; MVPA, moderate–vigorous physical activity; TBFM, total body fat mass; VPA, vigorous physical activity. ^a^ Stepwise method (pairwise exclusion). ^b^ Enter method (pairwise exclusion). Variables excluded: Model 1: weight, BMI, BMI z-score, BFM, MM, and Trunk FM; Model 2: SBP, DBP, and VO_2_ peak (mL/min); Model 3: SBP and VO_2_ peak (mL/min); Model 4: Sed, LPA, MPA, and VPA; Model 5: Sed, LPA, MPA, and MVPA.

## References

[1] Reilly J.J., Kelly J. (2011). Long-term impact of overweight and obesity in childhood and adolescence on morbidity and premature mortality in adulthood: Systematic review. Int. J. Obes..

[2] Sahoo K., Sahoo B., Choudhury A.K., Sofi N.Y., Kumar R., Bhadoria A.S. (2015). Childhood obesity: Causes and consequences. J. Fam. Med. Prim. Care.

[3] Inchley J., Currie D., Jewell J., Breda J., Barnekow V. (2017). Adolescent Obesity and Related Behaviours: Trends and Inequalities in the WHO European Region, 2002–2014.

[4] Lee H., Pantazis A., Cheng P., Dennisuk L., Clarke P.J., Lee J.M. (2016). The Association Between Adolescent Obesity and Disability Incidence in Young Adulthood. J. Adolesc. Health.

[5] Sommer A., Twig G. (2018). The Impact of Childhood and Adolescent Obesity on Cardiovascular Risk in Adulthood: A Systematic Review. Curr. Diabetes Rep..

[6] Davis P.H., Dawson J.D., Riley W.A., Lauer R.M. (2001). Carotid intimal-medial thickness is related to cardiovascular risk factors measured from childhood through middle age: The Muscatine Study. Circulation.

[7] Atabek M.E., Pirgon O., Kivrak A.S. (2007). Evidence for association between insulin resistance and premature carotid atherosclerosis in childhood obesity. Pediatr. Res..

[8] Bauer M., Caviezel S., Teynor A., Erbel R., Mahabadi A.A., Schmidt-Trucksäss A. (2012). Carotid intima-media thickness as a biomarker of subclinical atherosclerosis. Swiss Med. Wkly..

[9] Hayman L.L., Meininger J.C., Daniels S.R., McCrindle B.W., Helden L., Ross J., Dennison B.A., Steinberger J., Williams C.L. (2007). Primary prevention of cardiovascular disease in nursing practice: Focus on children and youth: A scientific statement from the American Heart Association Committee on Atherosclerosis, Hypertension, and Obesity in Youth of the Council on Cardiovascular Disease in the Young, Council on Cardiovascular Nursing, Council on Epidemiology and Prevention, and Council on Nutrition, Physical Activity, and Metabolism. Circulation.

[10] Zhao M., López-Bermejo A., Caserta C.A., Medeiros C.C.M., Kollias A., Bassols J., Romeo E.L., Ramos T.D.A., Stergiou G.S., Yang L. (2018). Metabolically Healthy Obesity and High Carotid Intima-Media Thickness in Children and Adolescents: International Childhood Vascular Structure Evaluation Consortium. Diabetes Care.

[11] Beauloye V., Zech F., Tran H.T., Clapuyt P., Maes M., Brichard S.M. (2007). Determinants of early atherosclerosis in obese children and adolescents. J. Clin. Endocrinol. Metab..

[12] Doyon A., Kracht D., Bayazit A.K., Deveci M., Duzova A., Krmar R.T., Litwin M., Niemirska A., Oguz B., Schmidt B.M. (2013). Carotid artery intima-media thickness and distensibility in children and adolescents: Reference values and role of body dimensions. Hypertension.

[13] Fang J., Zhang J.P., Luo C.X., Yu X.M., Lv L.Q. (2010). Carotid Intima-media thickness in childhood and adolescent obesity relations to abdominal obesity, high triglyceride level and insulin resistance. Int. J. Med. Sci..

[14] Ascenso A., Palmeira A., Pedro L.M., Martins S., Fonseca H. (2016). Physical activity and cardiorespiratory fitness, but not sedentary behavior, are associated with carotid intima-media thickness in obese adolescents. Eur. J. Pediatr..

[15] Green D.J., Maiorana A., O’Driscoll G., Taylor R. (2004). Effect of exercise training on endothelium-derived nitric oxide function in humans. J. Physiol..

[16] Arnold C., Wenta D., Müller-Ehmsen J., Sreeram N., Graf C. (2010). Progenitor cell number is correlated to physical performance in obese children and young adolescents. Cardiol. Young.

[17] Padilla J., Simmons G.H., Bender S.B., Arce-Esquivel A.A., Whyte J.J., Laughlin M.H. (2011). Vascular Effects of Exercise: Endothelial Adaptations Beyond Active Muscle Beds. Physiology.

[18] Green D.J., Walsh J.H., Maiorana A., Best M.J., Taylor R.R., O’Driscoll J.G. (2003). Exercise-induced improvement in endothelial dysfunction is not mediated by changes in CV risk factors: Pooled analysis of diverse patient populations. Am. J. Physiol. Circ. Physiol..

[19] Farpour-Lambert N.J., Aggoun Y., Marchand L.M., Martin X.E., Herrmann F.R., Beghetti M. (2009). Physical activity reduces systemic blood pressure and improves early markers of atherosclerosis in pre-pubertal obese children. J. Am. Coll. Cardiol..

[20] Park J.H., Miyashita M., Kwon Y.C., Park H.T., Kim E.H., Park J.K., Park K.B., Yoon S.R., Chung J.W., Nakamura Y. (2012). A 12-week after-school physical activity programme improves endothelial cell function in overweight and obese children: A randomised controlled study. BMC Pediatr..

[21] de Onis M., Lobstein T. (2010). Defining obesity risk status in the general childhood population: Which cut-offs should we use?. Int. J. Pediatr. Obes..

[22] Videira-Silva A., Sardinha L., Fonseca H. (2018). Effect of a Physical Activity Consultation in the Management of Adolescent Overweight (the PAC-MAnO project): Study rationale, design and methods. BMJ Paediatr. Open.

[23] de Onis M., Onyango A.W., Borghi E., Siyam A., Nishida C., Siekmann J. (2007). Development of a WHO growth reference for school-aged children and adolescents. Bull. World Health Organ..

[24] NHANES (2007). Dual Energy X-ray Absorptiometry (DXA) Procedures Manual. http://www.cdc.gov/nchs/data/nhanes/nhanes_07_08/manual_dexa.pdf.

[25] Tanaka H., Monahan K.D., Seals D.R. (2001). Age-predicted maximal heart rate revisited. J. Am. Coll. Cardiol..

[26] Pescatello L., Arena R., Riebe D., Thompson P., ACSM (2013). ACSM’s Guidelines for Exercise Testing and Prescription.

[27] Freedson P., Pober D., Janz K.F. (2005). Calibration of accelerometer output for children. Med. Sci. Sports Exerc..

[28] Schulz K.F., Altman D.G., Moher D., Group C. (2010). CONSORT 2010 statement: Updated guidelines for reporting parallel group randomised trials. BMJ.

[29] Park M.H., Skow Á., De Matteis S., Kessel A.S., Saxena S., Viner R.M., Kinra S. (2015). Adiposity and carotid-intima media thickness in children and adolescents: A systematic review. BMC Pediatr..

[30] Gooty V.D., Sinaiko A.R., Ryder J.R., Dengel D.R., Jacobs D.R., Steinberger J. (2018). Association Between Carotid Intima Media Thickness, Age, and Cardiovascular Risk Factors in Children and Adolescents. Metab. Syndr. Relat. Disord..

[31] Hacihamdioğlu B., Okutan V., Yozgat Y., Yildirim D., Kocaoğlu M., Lenk M.K., Ozcan O. (2011). Abdominal obesity is an independent risk factor for increased carotid intima- media thickness in obese children. Turk. J. Pediatr..

[32] Melo X., Fernhall B., Santos D.A., Pinto R., Pimenta N.M., Sardinha L.B., Santa-Clara H. (2016). The acute effect of maximal exercise on central and peripheral arterial stiffness indices and hemodynamics in children and adults. Appl. Physiol. Nutr. Metab..

[33] Jiménez-Pavón D., Castillo M.J., Moreno L.A., Kafatos A., Manios Y., Kondaki K., Béghin L., Zaccaria M., de Henauw S., Widhalm K. (2011). Fitness and fatness are independently associated with markers of insulin resistance in European adolescents; the HELENA study. Int. J. Pediatr. Obes..

[34] Cali A.M., Caprio S. (2008). Obesity in children and adolescents. J. Clin. Endocrinol. Metab..

[35] Huang K., Zou C.C., Yang X.Z., Chen X.Q., Liang L. (2010). Carotid intima-media thickness and serum endothelial marker levels in obese children with metabolic syndrome. Arch. Pediatr. Adolesc. Med..

[36] Iannuzzi A., Licenziati M.R., Acampora C., Salvatore V., Auriemma L., Romano M.L., Panico S., Rubba P., Trevisan M. (2004). Increased carotid intima-media thickness and stiffness in obese children. Diabetes Care.

[37] Videira-Silva A., Fonseca H. (2017). The effect of a physical activity consultation on body mass index z-score of overweight adolescents: Results from a pediatric outpatient obesity clinic. Eur. J. Pediatr..

[38] Meyer A.A., Kundt G., Lenschow U., Schuff-Werner P., Kienast W. (2006). Improvement of early vascular changes and cardiovascular risk factors in obese children after a six-month exercise program. J. Am. Coll. Cardiol..

[39] Prior B.M., Yang H.T., Terjung R.L. (2004). What makes vessels grow with exercise training?. J. Appl. Physiol..

[40] Freedman D.S., Khan L.K., Dietz W.H., Srinivasan S.R., Berenson G.S. (2001). Relationship of childhood obesity to coronary heart disease risk factors in adulthood: The Bogalusa Heart Study. Pediatrics.

[41] Freedman D.S., Dietz W.H., Tang R., Mensah G.A., Bond M.G., Urbina E.M., Srinivasan S., Berenson G.S. (2004). The relation of obesity throughout life to carotid intima-media thickness in adulthood: The Bogalusa Heart Study. Int. J. Obes. Relat. Metab. Disord..

[42] Elkiran O., Yilmaz E., Koc M., Kamanli A., Ustundag B., Ilhan N. (2013). The association between intima media thickness, central obesity and diastolic blood pressure in obese and overweight children: A cross-sectional school-based study. Int. J. Cardiol..

[43] Hardy R., Maddock J., Ghosh A.K., Hughes A.D., Kuh D. (2019). The relationship between pubertal timing and markers of vascular and cardiac structure and function in men and women aged 60–64 years. Sci. Rep..

[44] Teran-Garcia M., Rankinen T., Bouchard C. (2008). Genes, exercise, growth, and the sedentary, obese child. J. Appl. Physiol..

